# Mobile Phone Support for Diabetes Self-Care Among Diverse Adults: Protocol for a Three-Arm Randomized Controlled Trial

**DOI:** 10.2196/resprot.9443

**Published:** 2018-04-10

**Authors:** Lyndsay A Nelson, Kenneth A Wallston, Sunil Kripalani, Robert A Greevy Jr, Tom A Elasy, Erin M Bergner, Chad K Gentry, Lindsay S Mayberry

**Affiliations:** ^1^ Center for Health Behavior and Health Education Department of Medicine Vanderbilt University Medical Center Nashville, TN United States; ^2^ Center for Diabetes Translation Research Department of Medicine Vanderbilt University Medical Center Nashville, TN United States; ^3^ School of Nursing Vanderbilt University Nashville, TN United States; ^4^ Center for Effective Health Communication Department of Medicine Vanderbilt University Medical Center Nashville, TN United States; ^5^ Center for Clinical Quality and Implementation Research Department of Medicine Vanderbilt University Medical Center Nashville, TN United States; ^6^ Department of Biostatistics Vanderbilt University Medical Center Nashville, TN United States; ^7^ Department of Pharmacy College of Pharmacy and Health Sciences Lipscomb University Nashville, TN United States

**Keywords:** mobile health, medication adherence, type 2 diabetes, text messaging, self-care, glycated hemoglobin

## Abstract

**Background:**

Nonadherence to self-care is common among patients with type 2 diabetes (T2D) and often leads to severe complications. Moreover, patients with T2D who have low socioeconomic status and are racial/ethnic minorities disproportionately experience barriers to adherence and poor outcomes. Basic phone technology (text messages and phone calls) provides a practical medium for delivering content to address patients’ barriers to adherence; however, trials are needed to explore long-term and sustainable effects of mobile phone interventions among diverse patients.

**Objective:**

The aim of this study is to evaluate the effects of mobile phone–based diabetes support interventions on self-care and hemoglobin A_1c_ (HbA_1c_) among adults with T2D using a 3-arm, 15-month randomized controlled trial with a Type 1 hybrid effectiveness-implementation approach. The intervention arms are (1) Rapid Encouragement/Education And Communications for Health (REACH) and (2) REACH + Family-focused Add-on for Motivating Self-care (FAMS).

**Methods:**

We recruited primary care patients with T2D (N=512) from Federally Qualified Health Centers and an academic medical center, prioritizing recruitment of publicly insured and minority patients from the latter. Eligible patients were prescribed daily diabetes medication and owned a cell phone with text messaging capability. We excluded patients whose most recent HbA_1c_ result within 12 months was <6.8% to support detection of intervention effects on HbA_1c_. Participants were randomly assigned to REACH only, REACH + FAMS, or the control condition. REACH provides text messages tailored to address patient-specific barriers to medication adherence based on the Information-Motivation-Behavioral skills model, whereas FAMS provides monthly phone coaching with related text message content focused on family and friend barriers to diet and exercise adherence. We collect HbA_1c_ and self-reported survey data at baseline and at 3, 6, and 12 months, and again at 15 months to assess sustained changes. We will use generalized estimating equation models to test the effects of REACH (either intervention arm) on HbA_1c_ relative to the control group, the potential additive effects of FAMS, and effects of either intervention on adherence to self-care behaviors and diabetes self-efficacy.

**Results:**

The trial is ongoing; recruitment closed December 2017. We plan to perform analyses on 6-month outcomes for FAMS in July 2018, and project to have 15-month data for REACH analyses in April 2019.

**Conclusions:**

Our study will be one of the first to evaluate a long-term, theory-based text messaging intervention to promote self-care adherence among racially/ethnically and socioeconomically diverse adults with T2D. Moreover, our study will assess the feasibility of a family-focused intervention delivered via mobile phones and compare the effects of text messaging alone versus text messaging plus phone coaching. Findings will advance our understanding of how interventions delivered by phone can benefit diverse patients with chronic conditions.

**Trial Registration:**

ClinicalTrials.gov NCT02409329; https://clinicaltrials.gov/ct2/show/NCT02409329 (Archived by WebCite at http://www.webcitation.org/6yHkg9SSl); NCT02481596; https://clinicaltrials.gov/ct2/show/NCT02481596 (Archived by WebCite at http://www.webcitation.org/6yHkj9XD4)

## Introduction

### Background

The prevalence of diabetes is rapidly rising at both a global [1] and national level [2]. Individuals with diabetes are at a higher risk of heart disease, stroke, kidney disease, and premature mortality [1,3-5]. Type 2 diabetes (T2D) can be managed and its complications avoided by engaging in self-care, including healthy diet, exercise, self-monitoring of blood glucose (SMBG), and taking medications as prescribed [6]. However, multiple barriers impede self-care adherence for patients with T2D [7-10]. Racial/ethnic minorities and people with low socioeconomic status (SES) tend to experience more barriers to diabetes self-care [11,12] and, in turn, have worse self-care adherence [13,14], more complications [13,15], and worse glycemic control (ie, hemoglobin A_1c_ [HbA_1c_]) [16,17].

Basic mobile phone technology (text messaging and phone calls) presents viable opportunities to reach and support adults with T2D to improve self-care adherence and HbA_1c_ [18-20]. The vast majority of American adults (95%) own cell phones [21]; however, non-whites and those with less education and income are less likely to own a smartphone [21]. Text messaging and phone calls do not require a smartphone, and text messaging is the most common cell phone activity among all mobile phone users [22]. This ubiquity suggests potential to reach patients with low SES and racially/ethnically diverse patients [23,24]. Moreover, text messages can deliver tailored content and address modifiable barriers to diabetes self-care.

Involving human support as part of a diabetes mobile phone intervention may enhance efficacy [25,26] and improve participant engagement [27], particularly among disadvantaged or vulnerable patients [28]. In a recent 6-month randomized controlled trial (RCT), participants were assigned to receive health coaching along with access to a diabetes support app or only health coaching [29]. Although both groups had improved HbA_1c_ levels, the coaching group showed accelerated improvements [29]. A handful of other health promotion interventions in general populations have compared text messaging alone against text messaging plus human counselors, but the samples in these interventions have been small and therefore more research is needed [30].

In summary, adherence to diabetes self-care remains a prevalent problem and sustainable real-world solutions for diverse patients are needed [31]. Automated text messaging interventions can be resource- and cost-effective and have improved adherence and HbA_1c_ among underserved groups up to 6 months [32-34]; however, few have been evaluated in long-term trials with diverse samples [35]. Furthermore, none to our knowledge have assessed sustainability of effects after text messaging ends. Finally, it remains unclear whether a human coach or educator in concert with automated text messaging would be more effective for improving diabetes outcomes than text messaging alone.

### Objective

In response to these gaps in knowledge, we are conducting a 3-arm RCT to evaluate the effects of mobile phone–based diabetes self-care support interventions on self-care adherence and HbA_1c_ among adults with T2D who are diverse with respect to SES and race or ethnicity. The trial consists of 2 intervention arms and a control group. Intervention arms are (1) Rapid Encouragement/Education And Communications for Health (REACH) and (2) REACH + Family-focused Add-on for Motivating Self-care (FAMS). Both interventions were previously developed and tested for usability among racially/ethnically diverse and predominantly low-SES samples recruited from Federally Qualified Health Centers (FQHCs) [36,37]. REACH provides text messages tailored to address patient-specific barriers to medication adherence based on the Information-Motivation-Behavioral skills (IMB) model [38,39], whereas FAMS provides monthly phone coaching with related text message content focused on family and friend barriers to diet and exercise adherence [37].

The study is designed to evaluate the effects of REACH (either intervention arm) on HbA_1c_ relative to the control group, while assessing the additive effects of FAMS and effects of either intervention on adherence to self-care behaviors and diabetes self-efficacy. We will also explore the effects of each intervention arm on the psychosocial mechanisms targeted by each intervention and effect modification by race/ethnicity and SES.

## Methods

### Study Design

**Figure 1:**
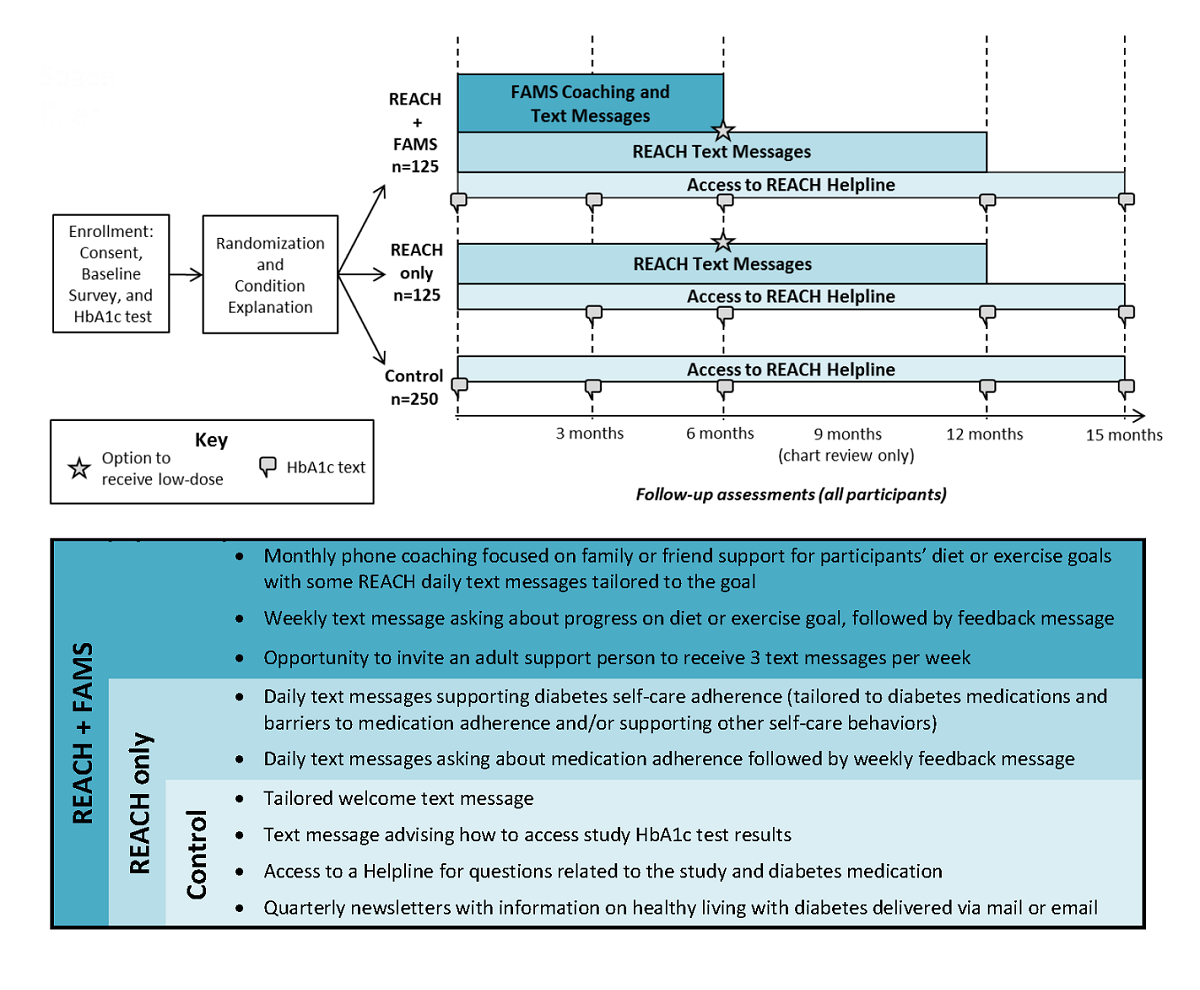
Top panel: Rapid Encouragement/Education And Communications for Health (REACH) randomized controlled trial design. Participants are randomized to REACH + Family-focused Add-on for Motivating Self-care (FAMS), REACH only, or the control condition. Bottom panel: Components received by each condition. Components are cumulative (eg, all participants receive control components). HbA_1c_: hemoglobin A_1c_.

We are conducting a 15-month, 3-arm RCT with 2 treatment arms and 1 control arm. We are using an effectiveness-implementation hybrid design to evaluate the effectiveness of the interventions while planning for and collecting information about implementation potential (Type 1 approach) [40]. This paper focuses primarily on the protocol for evaluating effectiveness, but REACH was designed to be sustainable [36], and our community-based research methods lay the groundwork to explore barriers and facilitators to implementation in FQHCs (briefly described in the Discussion section). For the trial, interested and eligible patients with T2D were recruited from primary care clinics. We designed our recruitment approach to overrepresent racial/ethnic minorities and patients with low SES. Participants in each arm complete study measures at baseline and 3, 6, 12, and 15 months post baseline (Figure 1, top panel). Participants in either intervention arm receive intervention exposure for 12 months; sustained changes are assessed with a 15-month follow-up.

#### Recruitment and Eligibility

We recruited participants across clinic sites in and around Nashville, Tennessee, including 13 FQHC locations and 3 Vanderbilt University Medical Center primary care locations. Recruitment strategies included the use of flyers, interest cards, referrals from clinic staff, mailing opt-in or opt-out letters (depending on clinic preference) to patients identified through the electronic health record (EHR) with follow-up calls, and in-person contact with patients in clinic waiting rooms or at clinic and community events. We oversampled patients who are racial/ethnic minorities and those who have low SES in several ways. First, our goal was to recruit at least 200 participants from FQHCs which serve uninsured or underinsured patients.

Second, when recruiting from Vanderbilt clinics, we prioritized the recruitment of patients with public health insurance (eg, TennCare [Medicaid], Medicare) only and/or who were racial/ethnic minorities.

Eligible participants were at least 18 years of age, had a diagnosis of T2D (both self-reported and confirmed either in the EHR or by a provider), were currently prescribed a daily diabetes medication (oral, insulin, and/or noninsulin injectables) and responsible for taking their diabetes medications (ie, without assistance from a caregiver), owned a cell phone with text messaging capability, received care at one of the participating clinics, and could speak and read in English. We excluded participants whose most recent HbA_1c_ value within 12 months was <6.8% to ensure room to lower HbA_1c_ and detect intervention effects (ie, avoid floor effects). In addition, because participants assigned to FAMS receive phone coaching, we excluded patients who had auditory limitations or an inability to orally communicate, as determined by trained research assistants (RAs). Patients who failed a brief cognitive screener [41] were excluded to help ensure accuracy of the measures and data integrity.

Finally, because all participants receive and are asked to interact using text messages, we excluded patients who were unable to receive, read or send text messages after demonstration by an RA (some participants with visual limitations were able to text and were therefore enrolled). We did not exclude participants based on comorbidities.

#### Data and Procedures

RAs met with interested patients in a private room at their respective clinics to verify eligibility, administer informed consent, and administer survey instruments. Most baseline surveys were administered aloud during the in-person meeting with the RA in a private room at the clinic. Less frequently, we consented patients over the phone and then mailed a copy of the consent and survey or emailed a link to sign the consent form and complete the survey via the Web (based on participant preference).

Participants have options on how to complete follow-up surveys, although we encourage in-person appointments in general, particularly for participants who may have trouble completing study materials independently due to limited health literacy or visual acuity difficulties. Survey completion can occur in one of 4 ways: (1) in-person with an RA at the participant’s clinic, (2) independently using paper surveys, (3) independently using online surveys, or (4) by phone with an RA. For in-person appointments, we aim to schedule the study appointment on the same day as the patient’s clinic appointment to make participation more convenient, and we try to align future clinic HbA_1c_ tests with follow-up study appointments.

Unless participants have had an HbA_1c_ test within the past 3 weeks or one is scheduled for the day of a study appointment, we either request that their provider order a lab-drawn HbA_1c_ test or ask participants to complete a mail-in HbA_1c_ test kit [42,43], depending on clinic preference. Mail-in kits contain all the necessary supplies to collect a sample of blood using a finger stick onto specialty paper (General Electric Health care) which is then mailed to the laboratory for dried blood spot analysis. Each kit is deidentified and linked to a unique barcode ID label. CoreMedica Laboratories (Lees Summit, Missouri), a specialty reference laboratory accredited by the College of American Pathologists, provides kits, analyzes the samples, and sends us the results.

RAs enter participants’ responses to survey questions into Research Electronic Data Capture (REDCap; Nashville, TN), a secure, Web-based application developed at Vanderbilt and designed to support data capture for multisite studies [44]. RAs access patient participants’ EHRs or clinics send us EHR data for enrolled participants, depending on clinic preference. EHR data are used to confirm and collect the type and quantity of prescribed diabetes medication and to collect results of clinic-administered HbA_1c_ tests. Participants’ relevant survey responses, HbA_1c_ results, and EHR data are transferred from REDCap to a digital health platform called MEMOTEXT (Bethesda, MD), via an application programming interface. MEMOTEXT uses participant information to schedule text message delivery and to tailor and send text messages to participants. Survey procedures, HbA_1c_ test procedures, and EHR reviews are repeated at each assessment (3, 6, 12, and 15 months), and text message content tailoring is updated by MEMOTEXT to reflect most current data. Additionally, we conduct EHR reviews to collect participants’ HbA_1c_ results at 9 months if a result is available.

### Measures

The same study measures are administered to all participants, regardless of condition. The schedule of measures is shown in Table 1. In the section below we focus on those measures central to the analyses outlined in this paper.

#### Outcomes

The primary outcome is HbA_1c_. Secondary outcomes include adherence to diabetes medication, self-care (diet, exercise, and SMBG), and diabetes self-efficacy. We assess diabetes medication adherence with 2 validated self-report measures: (1) the Adherence to Refills and Medications Scale for Diabetes (ARMS-D) [45] and (2) the Summary of Diabetes Self-Care Activities medications subscale (SDSCA-MS) [46]. We ask the SDSCA-MS questions for each prescribed medication, separately, and average responses across medications [45]. The SDSCA-MS is a commonly used and widely accepted measure of diabetes medication adherence [57] which asks about number of days adherent, whereas the ARMS-D is a more sensitive measure that asks about perceived frequency of nonadherence, and is a stronger predictor of HbA_1c_ [45]. Currently, there is not an ideal self-report measure of medication adherence. All available measures have limitations, so using multiple medication adherence measures is recommended [58,59].

Healthy diet is assessed with 2 subscales from the Personal Diabetes Questionnaire that assess Problem Eating Behavior and Use of Information for Diet Decision Making [47]. Exercise is assessed with the short form of the International Physical Activity Questionnaire [48,49], which provides information on the time spent walking, in vigorous and moderate intensity activities, and in sedentary activities. SMBG is assessed using the SDSCA blood glucose testing subscale [46]. Finally, self-efficacy is assessed with the Perceived Diabetes Self-Management Scale [50].

**Table 1:** Study measures across time points.

Construct		Description, example, scale	Baseline	Follow-ups (months after baseline)			
				3	6	12	15
**Primary outcome**							
	Hemoglobin A_1c_	Result from lab-drawn clinic test or mail-in test kit	X	X	X	X	X^a^
**Secondary outcomes**							
	Medication adherence	Adherence to Refills and Medications Scale for Diabetes [45]; Summary of Diabetes Self-Care Activities medications subscale (SDSCA-MS) [46]	X	X	X	X	X
	Diet adherence	Personal Diabetes Questionnaire subscales for Problem Eating Behavior and Use of Information for Diet Decision Making [47]	X	X	X	X	X
	Exercise adherence	International Physical Activity Questionnaire–short form [48,49]	X	X	X	X	X
	Self-monitoring of blood glucose (SMBG) adherence	SDSCA–SMBG subscale [46]	X	X	X	X	X
	Diabetes self-efficacy	Perceived Diabetes Self-Management Scale [50]	X	X	X	X	
**Mediators**							
	Barriers to diabetes medication adherence	Information, motivation, and behavioral skills-based barriers to medication adherence [36]	X	X	X	X	
	Family behaviors	Frequency of family or friends’ helpful and harmful behaviors over the past month	X	X	X	X	
**Moderators**							
	Race and ethnicity	White, African American, Asian, American Indian or Alaskan Native, Native Hawaiian or Pacific Islander, and/or other race; Hispanic or Latino or not Hispanic or Latino	X				
	Education	Years of school completed	X				
	Income	Total household income in 1 year	X			X	
	Insurance status	Uninsured, private, or public	X				
**Other measures**							
	Other sociodemographics	Gender, age, marital status, living situation	X				
	Diabetes characteristics	Insulin status, number of prescribed diabetes medications	X	X	X	X	X
	Mobile phone use	Use of smartphones and health apps, frequency of text messaging, and frequency of not being able to text and/or call because of reaching monthly limits	X			X	
	Depression	Patient Health Questionnaire–8 [51]	X	X	X	X	
	Health literacy	Brief Health Literacy Screen [52]	X				
	Numeracy	Subjective Numeracy Scale [53]	X				
	Sociological stressors	Tool for Assessing Patients’ Stressors [54]	X			X	
	Trait self-control	Brief Self-Control Scale (8-item subset) [55]	X		X		
	Diabetes duration	Length of time diagnosed with type 2 diabetes	X				
	Emergency room (ER) visits and Hospitalizations	Number of times in ER and hospitalizations in the last year	X			X	
	Smoking status	Behavioral Risk Factor Surveillance System items on tobacco use [56]	X	X	X	X	
	Alcohol consumption	Frequency of having a drink containing alcohol	X	X	X	X	

^a^We will also review medical charts at 9 months to collect HbA_1c_ values for those participants who have this data available since there is no planned follow-up assessment at this time point.

#### Mediators and Moderators

We also evaluate hypothesized mediators targeted by each of the interventions and moderators of intervention effects. REACH seeks to improve medication adherence via reductions in personalized information, motivation, and behavioral skills barriers identified by study assessments. We measure participants’ information, motivation, and behavioral skills-based barriers to adherence with an assessment developed for this trial, which maps barriers to diabetes medication adherence onto the IMB model constructs [36]. There are 31 barriers plus 5 insulin-specific barriers for participants who were prescribed insulin. To complete the measure, participants first indicate whether each barrier either “Sometimes” or “Never” applies to them. Next, for the barriers rated as “Sometimes,” participants rate the degree to which the barrier applies to them from 1=“a little” to 10=“a lot.” The purpose of this measure is (1) to identify REACH participants’ barriers to diabetes medication adherence so text message content can be tailored to their 4 highest rated barriers and (2) to ascertain whether the REACH intervention reduced participants’ barrier scores (relative to the control group) and whether changes in these barriers drove changes in diabetes medication adherence or HbA_1c_.

FAMS targets diabetes-specific helpful and harmful behaviors from family and friends. To measure these behaviors, we use a measure developed for this trial which assesses the frequency with which participants’ family or friends performed helpful and harmful behaviors over the past month. Example items are “How often do your family members… exercise with you or ask you to exercise with them?” (helpful item) or “…argue with you about your food choices or your health?” (harmful item), with response options on a scale from 1=“never in the past month” to 5=“twice or more each week.” Helpful and harmful items are averaged separately to produce 2 scores ranging from 1 to 5 with higher scores indicating more helpful or harmful family involvement in the patients’ diabetes self-care, respectively.

Finally, we plan to explore differential intervention effects based on participants’ race/ethnicity and SES (ie, income, insurance type, and education). As described above, racial/ethnic minorities and persons with low SES who have T2D tend to have worse self-care adherence and HbA_1c_ [13,14,16,17]; therefore, we anticipate these participants will experience more benefit from the intervention compared with participants who are white or have high SES. Each of these variables will be assessed with self-report at baseline.

### Randomization

During enrollment, RAs explain to participants that all study participants receive a mobile phone–based program with different types and frequencies of text messages and phone calls. RAs also tell participants that a member of the research team will call them in a few days to explain more about what to expect based on their assigned condition. After enrollment, participants are randomized to one of the 3 arms using optimal multivariate matching to ensure better balance in the primary outcome and important covariates across arms [60]. The variables we use to match participants include baseline HbA_1c_, insulin status, race, age, duration of diabetes, gender, income, and education. We give diabetes duration (rank value to correct for skew) and HbA_1c_ greater weight [61]. Twice as many participants are assigned to the control condition (n=250) as those assigned to REACH only (n=125) or REACH + FAMS (n=125). To accommodate this 2:1:1 design, patients are matched and then randomized to control or REACH and those randomized to REACH are matched and randomized to REACH only or REACH + FAMS. This helps ensure covariate balance among all 3 arms. For those assigned to REACH + FAMS, the FAMS intervention components last for the first 6 months only. All participants in the intervention arms receive REACH only for the latter 6 months of the exposure period (Figure 1, top panel).

Within a week of enrollment, participants are randomized, and a member of the research team calls each participant to explain what to expect from the mobile phone program to which they are assigned and obtain any information needed specifically for their assigned condition (eg, preferred times to receive daily text messages if assigned to either intervention arm). If we are unable to reach participants for this condition explanation within 3 weeks, they are administratively withdrawn; we still include these participants’ baseline data in our analyses but discontinue attempts to contact them. This run-in period ensures that the initiation of the study experience aligns with baseline data and identifies individuals who may be difficult to contact and therefore not good candidates for the 15-month trial. During the condition explanation we reiterate and assess participants’ understanding of the intervention components available to them, based on their condition. We do not use the terms “intervention” or “control” to explain the assigned conditions. Each condition is described briefly below and in Figure 1, bottom panel; the intervention components are described in more detail in the respective development papers for REACH [36] and FAMS [37].

#### Control

Participants assigned to the control condition maintain care as usual (ie, medication treatment and physician monitoring) but also receive a welcome text message following enrollment, as well as a text message advising how to access their study HbA_1c_ test result following enrollment and each completed follow-up. Control participants also receive access to the REACH Helpline (for questions related to the study and diabetes medications) and receive quarterly newsletters with information on healthy living with diabetes. Providing support and resources to the control group was important for our partnerships with clinics and an ethical decision because of our goal to oversample patients who were at risk (eg, high HbA_1c_ and patients with low SES). We provided these same resources to participants in all arms.

#### REACH Only

Participants assigned to REACH only receive all the components that control participants receive, plus the REACH text messages. REACH messages include daily messages promoting self-care, including tailored messages to address user-specific barriers to medication adherence based on responses to the IMB barrier assessment, nontailored text messages addressing other self-care behaviors, daily messages asking about the participant’s medication adherence for the day, and weekly feedback messages on his or her adherence. After 6 months, participants have the option to receive fewer text messages for the remaining 6 months of the intervention (ie, the “low-dose” option). The ideal frequency or dose of text messages for improving outcomes in an intervention is unclear [62]; we included the low-dose option to sustain engagement among participants who may prefer fewer-than-daily text messages. In a recent meta-analysis, there was no difference in chronic disease medication adherence between interventions using daily text messages and those using less frequent messaging [20]. Other evidence suggests that decreasing the frequency of texts over time or allowing users to choose their desired frequency is more efficacious than applying predetermined fixed or varying frequencies [30]. REACH participants who choose the low-dose option receive 3 or 4 self-care promotion messages each week and 1 message asking about medication adherence each week followed by feedback on their adherence.

#### REACH + FAMS

Participants assigned to REACH + FAMS receive all the components delivered to the aforementioned conditions, plus additional intervention components for the first 6 months. FAMS components include monthly phone coaching with counselors or health coaches (established or in-training; ie, persons with experience using basic helping skills who have also been trained in the FAMS protocols). During coaching, participants set healthy diet and exercise goals, and work with coaches to improve their ability to manage family or friends' actions that might support or interfere with the goal. Text messages tailored to the goal set during coaching replace the nontailored diet and exercise messages in REACH. FAMS participants can adjust the goal or set a new goal during each coaching session and have the opportunity to invite a family member or friend to receive text messages as a support person at any point during the first 6 months. The support person text message content aims to help enrolled support persons to be thoughtful about providing support and to initiate conversations with the participant about his or her diabetes and self-care goals. After 6 months, the FAMS components end, participants are offered the low-dose option described above, and they continue to receive REACH text messages for the next 6 months.

### Treatment Fidelity

We have implemented several fidelity checks to ensure that participants receive the interventions as intended. First, text messages are automated to help ensure users have the intended experience. Second, we monitor text messages to identify and correct errors and make contact with participants who stop responding to address any technical issues. MEMOTEXT securely collects and stores all text message data (eg, date and time text messages are sent and received, participants’ text message responses). Our team performs weekly checks on these data to ensure the text messages are delivered and monitor participants’ responses. As part of the REACH intervention, participants receive a daily adherence text message that asks them whether they have taken all of their diabetes medication that day. Participants are asked to reply either Yes or No. If they answer No, participants receive a follow-up text message asking them to please tell us why, with several response options (ie, “1=forgot, 2=sick, 3=clinic told me to, 4=ran out of meds, or type out a reason”). We monitor responses to these messages weekly, and if a participant does not respond to any adherence text messages for 14 consecutive days, a team member will call the participant to determine whether he or she is having any technical problems. To avoid coercing participants into responding to text messages, we only ask whether they have had any problems lately with receiving or responding to their text messages. The date of the call and the participant’s response are documented, and we subsequently troubleshoot as needed. We do not make repeated calls if the participant remains nonresponsive but may contact a participant more than once if periods of consecutive nonresponse are separated by periods of responsiveness.

We also collect fidelity data on the FAMS coaching sessions. We track the number of FAMS phone coaching sessions completed by each participant and the content of each session. Fidelity data includes the goal set during coaching, the type of family or friend support or barrier discussed, the skill-building exercise employed, the verbal contract (eg, to implement a skill learnt during coaching, such as assertive communication, with a specific friend or family member), the participant’s confidence rating of his or her ability to complete the verbal contract, and, for subsequent sessions, the outcome of the verbal contract from the previous session. Fidelity data will be presented with results to inform the degree to which the intervention was delivered as intended and to provide context for interpretation of study findings. Fidelity data will also serve as a process benchmark for future trials that may seek to reproduce the study findings or implementation studies that engage clinic staff in intervention delivery.

### Statistical Analysis Plan

The study is designed to evaluate the effects of REACH (either intervention arm) on HbA_1c_ relative to the control group (primary analysis), while assessing the effects of FAMS. We will use generalized estimating equation models to estimate potentially time-varying intervention effects while adjusting for the baseline measure of the outcome and the type of HbA_1c_ test result (ie, lab-drawn at the clinic or by using the mail-in kit). The models use clustered data and allow nonlinear associations between baseline and follow-up outcome measures. A lag 1 autoregressive correlation structure will be used and alternative correlation structures tested to demonstrate the results are robust to model selection. We will use a longitudinal model to evaluate intervention effects. We will use an omnibus test for the intervention effect, then provide point-estimates with confidence intervals for each follow-up, and graphically depict our results.

Analysis will follow a conservative intention-to-treat principle, and participants with missing values will be included along with those with complete data. Multiple imputation will be used to impute missing covariate and outcome values. The analysis with multiple imputation assumes Missing-at-Random (ie, the model properly handles missing data by including covariates associated with reasons for dropout). A sensitivity analysis for the impact of the imputation of missing outcome data will exclude the outcome from the imputation process and analyze only the observed outcomes.

#### Primary Analysis

We will test the effects of receiving REACH on HbA_1c_ (primary outcome) and medication adherence (secondary outcome) compared with the control condition. This model will not distinguish between the REACH only and REACH + FAMS arms. We hypothesize participants assigned to REACH will experience greater improvements in medication adherence and HbA_1c_ than participants assigned to the control condition.

#### Secondary Analysis

In addition, we will test the effects of both intervention arms (REACH only and, separately, REACH + FAMS) on diet, exercise, SMBG, and diabetes self-efficacy relative to the control group. Finally, we will assess whether participants assigned to REACH + FAMS experience greater improvements in HbA_1c_, medication adherence, diet, exercise, SMBG, and self-efficacy compared with those assigned to REACH only.

#### Mediation and Moderation Analyses

We will conduct 2 separate mediation analyses, one for REACH (including participants in either REACH arm relative to the control arm) and one for FAMS (including participants in the REACH + FAMS arm relative to the control arm). REACH mediation analyses will examine whether REACH improves participants’ IMB barriers to diabetes medication adherence and whether such improvements explain REACH’s effect on adherence and/or HbA_1c_. FAMS mediation analyses will examine whether FAMS improves participants’ reported diabetes-specific helpful and harmful family and friend behaviors and whether such improvements explain REACH + FAMS effect on diet, exercise, and diabetes self-efficacy. We hypothesize that improvements in IMB barriers will drive improvements in medication adherence and HbA_1c_, and improvements in family and friend behaviors will drive effects on diet, exercise, and diabetes self-efficacy. Specifically, we will use between-person mediation analyses with latent change scores for mediators and outcomes [63,64], and we will use bootstrapping to obtain CIs for indirect effects [65]. Lastly, we will explore whether race/ethnicity, education, and income modify the intervention effects by adding interaction terms to models evaluating intervention effects.

#### Sample Size and Power

Our target sample was 500 patient participants and we ultimately enrolled 512. With an anticipated dropout rate of 20%, we will have at least 400 participants for analysis of intervention effects up to 15 months. Power calculations were performed using Power and Sample Size (PS) software (Nashville, TN) at 80% power for a 2-sided text (alpha=.05). Based on HbA_1c_ data from a prior study with 314 adult patients with T2D from a FQHC in Nashville, TN, we estimate the residual error from a model of HbA_1c_ will have a standard deviation ≤2% [45]. Thus, this study will have 80% power to detect a true effect of 0.56% on HbA_1c_ by REACH at any follow-up time point if we have 400 participants for analysis.

### Ethics and Informed Consent

All procedures have been reviewed and approved by the Vanderbilt University Institutional Review Board (IRB) and this trial is registered on ClinicalTrials.gov (see NCT02409329 and NCT02481596). All data collected from participants at each assessment period are stored on REDCap’s secure server. Any participant data sent to MEMOTEXT are deidentified and stored on their Health Insurance Portability and Accountability Act (HIPAA)-compliant secure server. In addition, all reporting of text message data by MEMOTEXT and all recorded REACH Helpline voicemails are stored on their HIPAA-compliant Web server, and only IRB-approved study staff can access these voicemail messages using a secure passcode. EHR data are shared with the study team according to the policies of each individual clinic.

We included specific language in the informed consent document outlining our processes for securing participants’ data. We described that REACH Helpline voicemail messages, information shared via text message, and all study forms would be assigned a study number with no personal identifying information and be either password-protected on a secure server or in a locked filing cabinet at Vanderbilt. We explained that research team members would only access personal information for necessary study procedures, such as to issue payment or contact for follow-up appointments. Finally, we explained to participants that, if they share or lose their phone, the study text messages may disclose to others that they have diabetes, take diabetes medications, and/or received an HbA_1c_ test. All participants were provided with the REACH Helpline number and encouraged to call to ask questions about the study.

## Results

**Figure 2:**
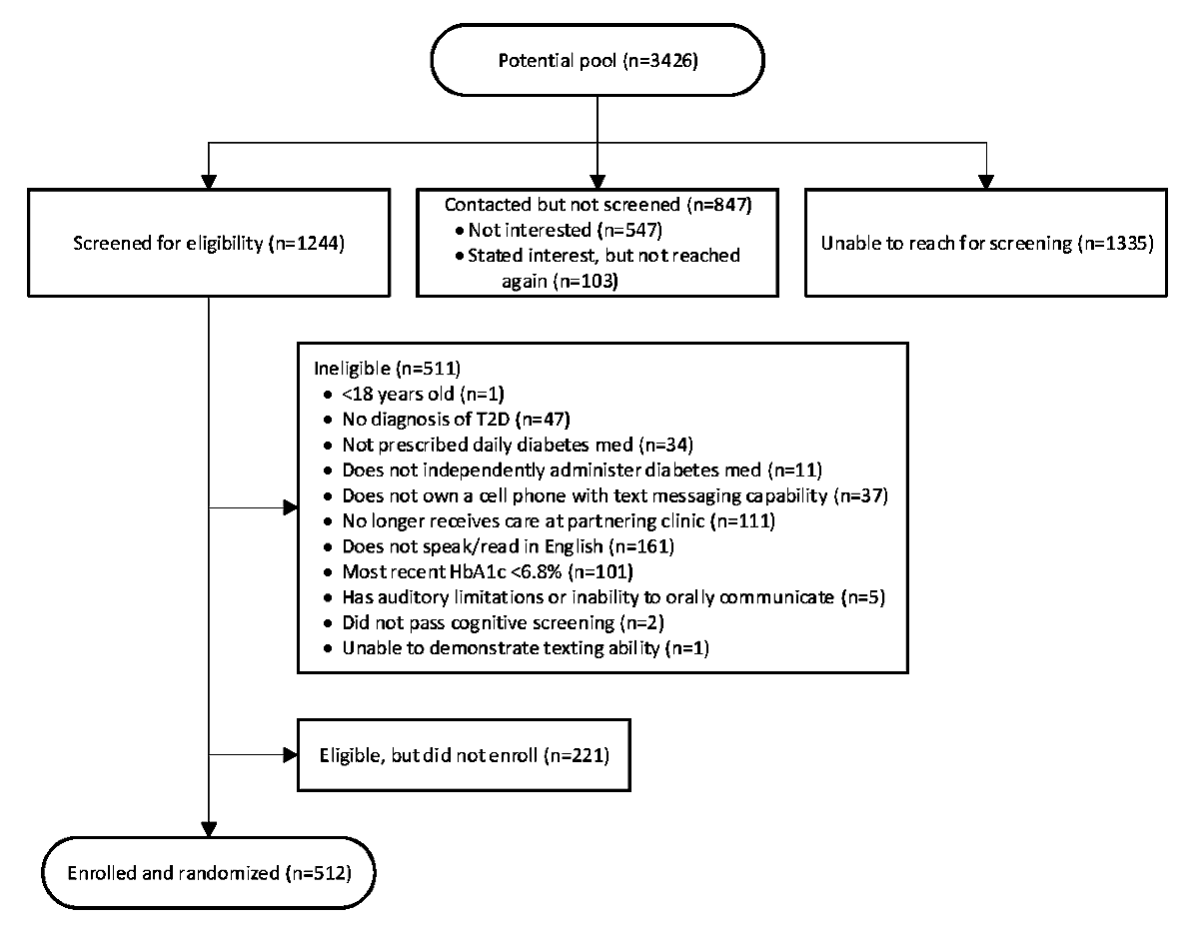
Flowchart of potential patient participants through study recruitment. HbA_1c_: hemoglobin A_1c_.

Recruitment began in May 2016 and ended in December 2017. Figure 2 shows recruitment results. Of the 3426 patients identified as potentially eligible throughout study recruitment, we were able to contact 61.03% (2091/3426) by phone or in person and screen 36.31% (1244/3426) for eligibility. Of those screened, 41.08% (511/1244) were ineligible and 41.16% (512/1244) enrolled. Most common reasons for ineligibility were not speaking or reading in English (31.5%, 161/511, of those ineligible), no longer receiving care at a partnering clinic (21.7%, 111/511), and having a most recent HbA_1c_ <6.8% (19.8%, 101/511). We administratively withdrew 6 participants or 1.2% (6/512) of those enrolled. Enrolled participants (N=512) have an average age of 56.0 (SD 9.5) years, and 54.1% (277/512) are female.

Approximately half (47.3%, 242/512) are non-Hispanic white, 39.4% (202/512) are non-Hispanic African American, 6.0% (31/512) are Hispanic, and 7.2% (37/512) reported being of other race and/or ethnicity (including multiracial). In addition, 41.7% (210/503) reported educational attainment of a high school degree or less, 55.8% (286/512) have annual incomes less than US $35,000, and 48.6% (247/508) are underinsured (23.2%, 118/508, have no insurance; 25.4%, 129/508, have public insurance only). About half (48.8%, 250/512) are taking insulin and average HbA_1c_ at baseline is 8.6% (SD 1.8%, median 8.2%, IQR 7.2%-9.6%). Most (98.6%, 493/500) baseline HbA_1c_ tests were taken within 30 days of study enrollment, and all were taken within 70 days of enrollment. As of this publication, we have at least 87% completion among participants through each follow-up assessment. We plan to perform analyses on 6-month outcomes for FAMS in July 2018 and have 15-month data for REACH analyses in April 2019.

## Discussion

### Principal Considerations

This study will be one of the first RCTs to deliver a long-term, theory-based, text messaging intervention to promote self-care adherence among racially/ethnically and socioeconomically diverse adults with T2D. We designed the interventions to use basic mobile phone technology (text messaging and phone calls) and provide an experience that is individually tailored and interactive for adult patients with T2D. We developed both interventions with input from racially diverse patients with low SES [36,37] and designed our recruitment strategies for the RCT to oversample racial/ethnic minorities and patients with fewer resources. Moreover, our study will explore the feasibility of a family-focused intervention delivered via mobile phones, and allows exploratory analyses comparing the effects of text messaging alone versus text messaging plus phone coaching. We will also be the first to provide information on the feasibility and acceptability of inviting members of a patients’ social support network to receive text messages about how to support the patient with his or her T2D, based on the 125 participants in our sample given the option to do so as part of FAMS.

Barriers to self-care adherence are personal, multidimensional, and change over time [66,67]. Findings from other studies suggest that helping patients overcome their unique barriers may improve adherence and HbA_1c_ [68]. For instance, in a 12-month RCT, intervention participants received phone calls from diabetes educators who provided tailored strategies for coping with self-care barriers [69]. HbA_1c_ decreased more among intervention participants than control participants, suggesting content addressing modifiable self-care barriers can be effective. However, study participants were predominantly white and well-educated [69], limiting the generalizability of the results. Not only will our diverse patient sample provide more generalizable results, but including measures that assess patients’ barriers to medication adherence and family and friend involvement in self-care will allow us to determine whether improvements in the psychosocial mechanisms targeted by the interventions explain improvements in outcomes.

Findings from the RCT will advance understanding of the health benefits of mobile phone–based interventions, with generalizability to racial/ethnic minorities and persons with low SES with chronic conditions such as diabetes [70,71]. The REACH intervention was designed to be incorporated into routine clinical care at FQHCs to support diabetes self-care adherence with minimal time investment from providers and staff. As a Type 1 effectiveness-implementation hybrid design, the primary focus of this study is to evaluate the intervention’s effectiveness. Therefore, we had research staff execute protocols to ensure a structured test of effects. For the secondary goal of assessing facilitators and barriers to implementation, we will invite intervention participants who have finished the trial, as well as FQHC providers and administrators, to participate in interviews to collect qualitative and quantitative data on their perceptions of REACH and FAMS. These interviews will focus on strategies for uptake and sustainability in clinic settings. This information will be used to develop recommendations for implementing and evaluating mobile phone–delivered interventions, like REACH and FAMS, in FQHC settings.

### Limitations

Limitations of this study include reliance on self-report measures of adherence. Compared with objective measures, self-report measures are subject to social desirability and recall bias. However, each measure of adherence has drawbacks. Self-report measures are inexpensive, brief, and unobtrusive, and we have selected validated measures with balancing strengths and weaknesses. Another challenge is participants changing their cell phone plans and numbers; however, the REACH Helpline (where participants can inform us of changes in their contact information), requesting secondary contact information (eg, a work number, a family member’s or friend’s number to use if we cannot reach them), calling participants after 14 consecutive days of nonresponse, and regular follow-ups help us maintain contact with participants. Our study is powered to examine the effects of receiving REACH on HbA_1c_; therefore, analyses examining the effects of other outcomes (ie, self-care behaviors, self-efficacy) and comparing the effects of either intervention arm are potentially very informative but may be underpowered. Because the trial does not include a separate FAMS condition (ie, without REACH), we are not able to evaluate the effects of FAMS only. Finally, the interventions are currently only available in English, which was necessary to enhance feasibility of successfully completing this initial trial; however, translation to Spanish is a goal, should they prove effective.

### Conclusions

We anticipate this study will help determine the effectiveness of a tailored text messaging intervention for supporting diabetes self-care adherence and reducing HbA_1c_ among racially/ethnically and socioeconomically diverse patients. Additionally, we aim to determine whether (1) tailoring IMB model-based content to user-specific medication adherence barriers is effective for improving medication adherence behavior and HbA_1c_, thereby supporting the IMB model as an appropriate framework for interventions to promote medication adherence in diabetes and (2) basic mobile phone technology is a feasible and potentially effective medium for family-focused interventions and for engaging family members and friends in adults’ self-care efforts. Beyond these primary aims, we will be able to examine data on users’ responses to text messages throughout the trial, the choice to receive fewer text messages after 6 months, and participant characteristics associated with either. Findings will inform the design and length of future text message–delivered interventions in similar populations.

## Abbreviations

ARMS-D: Adherence to Refills and Medications Scale for Diabetes
EHR: electronic health record
FAMS: Family-focused Add-on for Motivating Self-care
FQHC: Federally Qualified Health Center
HbA_1c_: hemoglobin A_1c_
HIPAA: Health Insurance Portability and Accountability Act
IMB: Information-Motivation-Behavioral Skills
IRB: institutional review board
RA: research assistant
RCT: randomized controlled trial
REACH: Rapid Encouragement/Education And Communications for Health
REDCap: Research Electronic Data Capture
SDSCA-MS: Summary of Diabetes Self-Care Activities medications subscale
SES: socioeconomic status
SMBG: Self-Monitoring of Blood Glucose
T2D: type 2 diabetes
